# A Bayesian approach for inducing sparsity in generalized linear models with multi-category response

**DOI:** 10.1186/1471-2105-16-S13-S13

**Published:** 2015-09-25

**Authors:** Behrouz Madahian, Sujoy Roy, Dale Bowman, Lih Y Deng, Ramin Homayouni

**Affiliations:** 1 Department of Mathematical Sciences, University of Memphis, Memphis, TN, USA; 2 Department of Biology, University of Memphis, Memphis, TN, USA; 3 Bioinformatics Program, University of Memphis, Memphis, TN, USA

**Keywords:** High Dimensional Data, Classification, Gibbs Sampling, Bayesian, Prostate Cancer

## Abstract

**Background:**

The dimension and complexity of high-throughput gene expression data create many challenges for downstream analysis. Several approaches exist to reduce the number of variables with respect to small sample sizes. In this study, we utilized the Generalized Double Pareto (GDP) prior to induce sparsity in a Bayesian Generalized Linear Model (GLM) setting. The approach was evaluated using a publicly available microarray dataset containing 99 samples corresponding to four different prostate cancer subtypes.

**Results:**

A hierarchical *S*parse *B*ayesian *G*LM using *G*DP prior (SBGG) was developed to take into account the progressive nature of the response variable. We obtained an average overall classification accuracy between 82.5% and 94%, which was higher than Support Vector Machine, Random Forest or a Sparse Bayesian GLM using double exponential priors. Additionally, SBGG outperforms the other 3 methods in correctly identifying pre-metastatic stages of cancer progression, which can prove extremely valuable for therapeutic and diagnostic purposes. Importantly, using Geneset Cohesion Analysis Tool, we found that the top 100 genes produced by SBGG had an average functional cohesion p-value of 2.0E-4 compared to 0.007 to 0.131 produced by the other methods.

**Conclusions:**

Using GDP in a Bayesian GLM model applied to cancer progression data results in better subclass prediction. In particular, the method identifies pre-metastatic stages of prostate cancer with substantially better accuracy and produces more functionally relevant gene sets.

## Introduction

Using high-throughput microarray or massively parallel RNA sequencing technologies, the expression levels of several thousand genes can be measured across a number of samples simultaneously. Analysis of gene expression data obtained by these technologies is mathematically challenging because generally the number of samples are small (usually tens to hundreds) compared to thousands of variables [1]. Several statistical methods in univariate analysis framework have been developed to address this problem [2-6]. However, single gene analysis is unable to identify weaker associations, especially for complex polygenic phenotypes for which the relevant variation is distributed across several genes [1]. In order to address these limitations, several approaches for simultaneous analysis of multiple variables have been developed [7-9]. These approaches require an initial feature selection method to identify a smaller set of genes with the strongest effect and discriminating power. Some variable selection methods in a regression framework include backward elimination, forward selection, and stepwise selection. One of the shortcomings of these methods is that they are discrete processes which are very sensitive to the changes in the data. That is, a minor change in data can result in very different models [10-12]. Additionally, the computational complexity of these methods, when the number of variables is very large make them less attractive for gene expression analysis [10,11]. Moreover in this setting, over-fitting is a major concern and may result in failure to identify important predictors. Thus, the data structure of typical gene expression experiments makes it difficult to use traditional multivariate regression analysis [1].

Several groups have developed methods to overcome drawbacks of multivariate regression analysis [7,8,10,12,13]. Various methods such as K-nearest neighbour classifiers [5], linear discriminant analysis [14], and classification trees [5] have been used for multi-class cancer classification and discovery [15-17]. However, gene selection and classification are treated as two separate steps which can limit their performance. One promising approach to analyse, predict, and classify binary or multi-category samples using gene expression data is Generalized Linear Models (GLM) [18-20]. However, due to the large number of variables, maximum likelihood estimates of parameters becomes computationally intensive and sometimes intractable. Additionally, since the sample size is much smaller than the number of variables, the maximum likelihood estimates may have large estimated variances and thus result in poor prediction accuracy. Finally, the maximization process may not converge to maximum likelihood estimates [8].

Previously, it was proposed that the prediction accuracy of GLMs can be improved by setting the parameters associated with unimportant variables to zero and thus obtaining more accurate prediction for the significant variables without over-fitting [11]. Least Absolute Shrinkage and Selection Operator (LASSO) is a well-known method for inducing sparseness in the model while highlighting the relevant variables [11,12,21]. Later, a Bayesian LASSO method was proposed by [22,23] in which double exponential prior is used on parameters in order to impose sparsity. However, these procedures may cause over-shrinkage of large coefficients due to the relatively light tails of the double exponential prior and introduce bias [24,25]. A modification of this approach, which uses normal-Jeffreys prior with heavier tails than double exponential distribution, is able to shrink small coefficients to zero while minimally shrinking large coefficients reducing bias in the model. However it has no meaning from an inferential aspect as it leads to an improper posterior [24]. An alternative class of hierarchical priors proposed in [15] uses Bayesian adaptive Lasso with non-convex penalization, but it lacks a simple analytic form. Others have proposed the Generalized Double Pareto (GDP) prior distribution, which has several advantages [24]. The GDP distribution has a spike at zero alongside studentt like tails. While GDP resembles double exponential density in the neighbourhood of zero, it has heavier tails compared to the double exponential, which remedies unwanted bias resulting from over shrinkage of parameters toward zero [24]. In addition, GDP has a simple analytic form and yields proper posteriors. In many of the approaches, the variables are assumed fixed, but in many cases where the predictor variables are random, such as gene expression data, assumptions can be made that result in the same formulation as in fixed case [26]. One such assumptions is a joint multivariate normal distribution for response and predictors, other is an analysis of response conditioned upon the random predictors.

In our previous work, we implemented a sparse Bayesian generalized linear model with double exponential prior to classify different subtypes of prostate cancer using gene expression profiles [27]. Given the limitations discussed above regarding this prior, in this study we aimed at using the GDP prior to overcome these issues. Here, we applied GDP for the first time into the Bayesian generalized linear model framework. The model was utilized to classify multi-category ordinal phenotypes based on gene expression data. We evaluated the model based on classification of progressive stages of prostate cancer using a publicly available microarray dataset [28]. Our specific objectives were to test if the model can: 1) result in a smaller subset of genes with high discriminating power, 2) obtain high classification accuracy; 3) identify more biologically relevant genes compared to other classification methods.

## Methods

Let ${\left[y_{i},\;{w_{i}}_{\text{1}},\;..,\;w_{ip}\right]}_{i=1}^{n}$ represent the dataset in which *y_i _*stands for response variable of the *i^th ^*subject with possible values 1, 2, 3*,..., k *where *k *is the number of categories of the ordinal response variable. In addition, let *w_ij _*represent the value of variable 'j' in sample 'i'. In the case of gene expression analysis, gene expression levels are measured for each sample and *w_ij _*represents expression level of gene *j *in *i^th ^*sample. We implemented GLM for ordinal response in Bayesian framework by utilizing logistic link function and careful introduction of latent variables [29]. In a Bayesian framework the joint distribution of all parameters is proportional to the likelihood multiplied by prior distributions on the parameters. This likelihood function for Bayesian Multinomial model is presented below. In this formula, *π_ij _*is the probability that *y_i _*equals *j *and *I*(*y_i _*= *j*) is an indicator function having value one if the sample i's response variable is in category *j *and zero otherwise. It should be noted that each sample contributes one value in the inner product to the equation below since the indicator function returns value of zero if *j *is not equal to the category of outcome for the sample.

$$L\left(\underset{-}{\pi }|\underset{-}{y}\right)=\overset{n}{\underset{i=1}{\prod }}\left[\overset{k}{\underset{i=1}{\prod }}\left[\pi_{ij}^{I\left(y_{i}=j\right)}\right]\right]$$

In order to be able to find the posterior distributions of parameters, we need to integrate the likelihood function multiplied by joint prior distributions of all parameters. However, this approach will result in an intractable integration. As explained in [29], in order to be able to set up the Gibbs sampler, we introduce 'n' independent latent variables *l*_1_*, l*_2_*, ..., l_n _*defined as $l_{i}=w_{i}^{T}\theta +e_{i}$. In this formula ***w***_*i *_is the vector of gene expressions for sample *i *defined as ***w***_*i *_= (*w*_*i*1_,..., *w_ip_*)*^T ^*and ***θ ***= (*θ*_1_,..., *θ_p_*)*^T ^*is the vector of parameters associated with gene 1 to gene *p*. We assume logistic distribuion on error temrs, $F\left(e_{i}\right)\;=\frac{1}{1+e^{-e_{i}}},$ to obtain logistic regression [30]. In order to be able to set up the Gibbs sampler, we approximate the logistic distribution on the latent variables with t-distribution defined as $l_{i}\sim t_{\upsilon }\left(w_{i}^{T}\theta \right)$. The reason for choosing t-distribution is that logistic distribution has heavy tails and normal distribution does not provide a good approximation [29,31]. Hence, we used the student-t distribution with  υ degrees of freedom on latent variables to provide a better approximation for the distribution on latent variables. We treat the degrees of freedom as unknown and estimate it alongside other parameters. It should be noted that this distribution is a non-central t-distribution with *v *degrees of freedom and non-centrality parameter $w_{i}^{T}\theta$. The following relationship is established between response and corresponding latent variable [29].

$$\begin{array}{c} y_{i}\text{ = }\left\{\begin{array}{ccc} 1 & iff & -\infty =\gamma_{1}\leq l_{i}<\gamma_{2}\\ 2 & iff & 0\;=\gamma_{\text{2}}\leq l_{i}<\gamma_{\text{3}}\\ : & & \\ k & iff & \gamma_{k}\leq l_{i}<{\gamma_{k}}_{+\text{1}}=\infty \end{array}\right) \end{array}$$

In order to insure that the thresholds are identifiable, following the guidelines of [29], we fix *γ*_2 _at zero and *γ*_1_, and *γ*_*k*+1 _are defined according to equation above. In the context of GLM, we use nonlinear link functions to associate the nonlinear, non-continuous response variable to the linear predictor $w_{i}^{T}\theta$. It should be noted that logistic distribution has heavy tails and thus normal distribution does not provide a good approximation and hence we used student-t distribution with  υ degrees of freedom on latent variables. We treat the degrees of freedom as unknown and estimate it alongside other parameters. Using the relations defined above, the probability of each sample being in category *j*(*j *= 1, 2*,..., k*) is derived in following equation in which *π_ij _*is the probability of sample i being from category *j *[29].

$$\zeta_{ij}=P\left(y_{i}\leq j\right)=P\left(l_{i}\leq {\gamma_{j}}_{+\text{1}}\right)=P\left(w_{i}^{T}\theta +e_{i}\leq {\gamma_{j}}_{+\text{1}}\right)=P\left(e_{i}\leq {\gamma_{j}}_{+\text{1}}-w_{i}^{T}\theta \right)=\frac{1}{1+e^{-\left(\gamma_{j+1}-w_{i}^{T}\theta \right)}};\;\pi_{ij}=\zeta_{ij}-\zeta_{ij-1}$$

In this way, the linear predictor $w_{i}^{T}\theta$is linked to the multi-category response variable *y_i_*. The function that links the linear predictor to the response variable is called a link function and in the multinomial Logistic model, this link function is cumulative distribution of a standard Logistic density as defined above [19,20,29]

### Prior distributions and Baysian set up

A sparse Bayesian ordinal logistic model was implemented which takes into account the ordinal nature of cancer progression stages and can accommodate a large number of variables. In order to sample *l_i _*from $t_{\upsilon }\left(w_{i}^{T}\theta \right)$, we use the following hierarchical model which is equivalent to sampling from the corresponding t-distribution [18]. This two-level hierarchical form is easier to work with both analytically and computationally compared to the original form of the t distribution [18].

$$l_{i}|{\text{Λ}}_{i},\theta \sim N\left({w_{i}}^{T}\theta ,{\frac{1}{\text{Λ}}}_{i}\right);\;{\text{Λ}}_{i}\sim Gamma\left(\frac{\upsilon }{2},\frac{\upsilon }{2}\right)$$

Here the gamma distribution is defined as $\pi \left(x|\alpha ,\;\beta \right)\;=\frac{\beta^{\alpha }}{\Gamma \left(\alpha \right)}x^{\alpha -\text{1}}e^{-\beta x}$. We put independent Generalized Double Pareto(GDP) priors on all *θ*s as defined in [24]. It should be noted that *θ_j _*is the parameter associated with gene *j*. This prior distribution has a spike at zero and light tails which enables us to incorporate sparsity in terms of number of variables used in the model [24].

$$f\left(\theta |\zeta ,\;\rho \right)\;=\;\frac{1}{\text{2}\zeta }*{\left(1+\frac{\left|\theta \right|}{\rho \zeta }\right)}^{-\left(\text{1}+\rho \right)};\rho ,\;\zeta >0$$

Letting $\theta_{j}\sim GDP\left(\zeta =\frac{\delta }{\rho },\;\rho \right)$ independently, the joint distribution of *θ*s is defined as follows.

$$\pi \left(\theta \right)=\overset{p}{\underset{j=1}{\prod }}\left[\frac{1}{2\frac{\delta }{\rho }}*{\left(\text{1 + }\frac{\left|\theta_{j}\right|}{\delta }\;\right)}^{-\left(\text{1}+\rho \right)}\right]\;$$

The GDP prior can be represented as a scale mixture of normal distributions leading to computational simplifications that makes Gibbs sampling feasible. The $GDP\left(\frac{\delta }{\rho },\rho \right)$ prior is equivalent to the following hierarchical representation [24].

$$\theta_{j}|\tau_{j}\sim N\left(0,\tau_{j}\right);\tau_{j}\sim Exp\left(\frac{\lambda_{j}^{2}}{2}\right);\lambda_{j}\sim Gamma\left(\rho ,\delta \right)$$

The hyper parameters *ρ *and *δ *control the shape of the GDP distribution and thus the amount of shrinkage induced. As *δ *increases, the distribution becomes flatter and variance increases. As *ρ *increases, the tails of distribution becomes lighter, variance becomes smaller, and the distribution becomes more peaked. Thus, large values of *ρ *may cause unwanted bias for large signals and stronger shrinkage for noise-like signals while larger values of *δ *flattens the distribution and we may lose the ability to shrink noise-like signals. As mentioned in [24], by increasing *ρ *and *δ *at the same rate the variance remains constant but tails of the distribution become lighter converging to the Laplace density in limit, leading to over-shrinkage of coefficients. In the absence of information on hyper parameters one can either set them to default values (*ρ *= *δ *= 1) or choose a hyper prior distribution and let data speak about the values of these hyper parameters. We adopt the following prior distributions for these parameters.

$$\pi \left(\rho \right)=\frac{c}{{\left(\text{1 }+c\rho \right)}^{\text{2}}};c>0\;\;\;\pi \left(\delta \right)=\frac{c\prime }{{\left(\text{1}+c\prime \delta \right)}^{\text{2}}};c\prime >0$$

The priors on *ρ *and *δ *correspond to generalized Pareto priors with location parameter 0, shape parameter 1, and scale parameters *c*^-1 ^and *c′*^-1 ^respectively. For sampling purposes, we do the following transformations that lead to uniform prior distribution for the new parameters [24].

$$u_{\text{1}}=\frac{1}{\text{1}+c\rho };\;\;\;u_{\text{2}}=\frac{1}{\text{1}+c\prime \delta }$$

**Figure 1:**
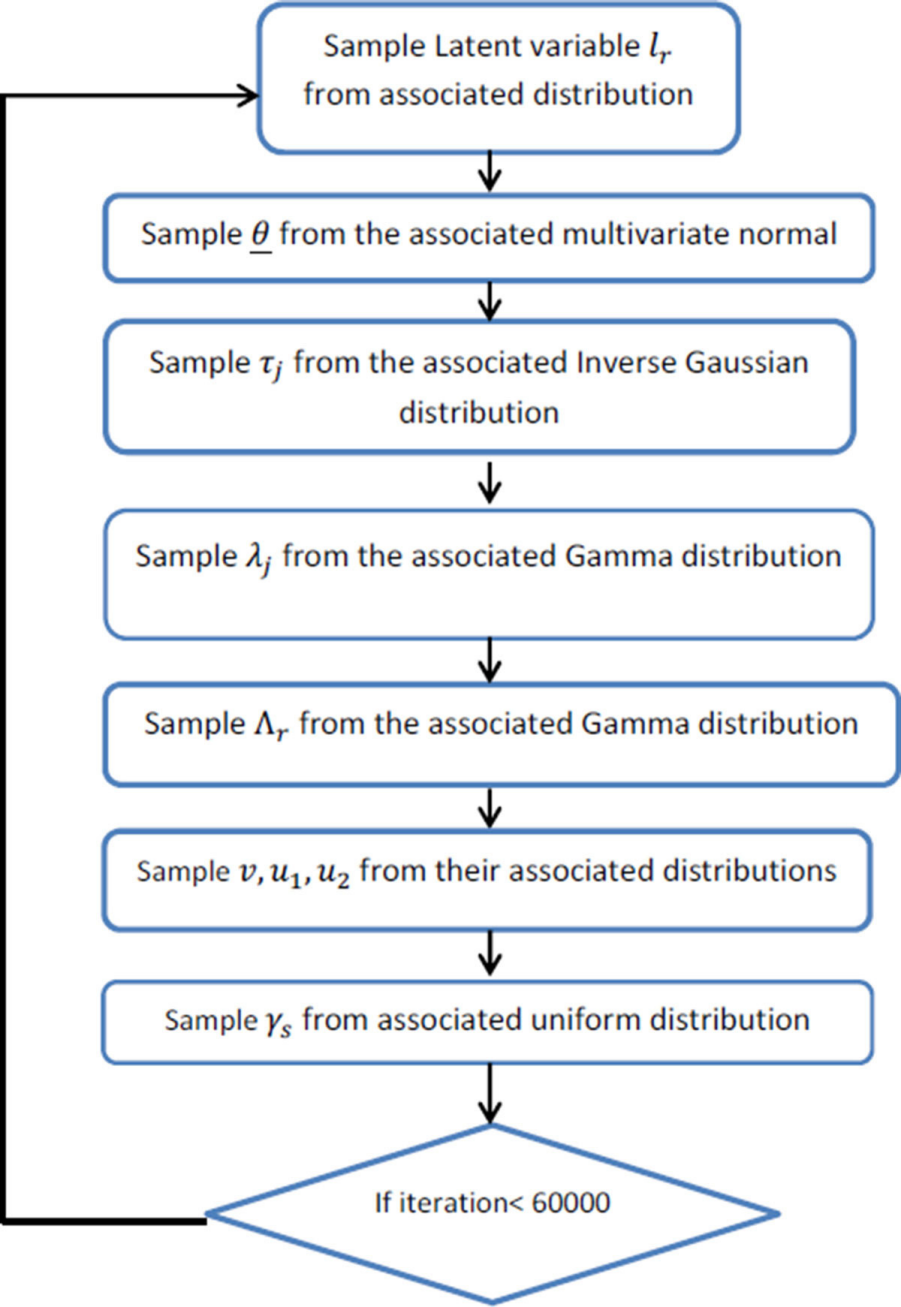
**Flow chart of Gibbs sampling procedure for SBGG**. Here j = 1, 2,..., p and r = 1, 2,..., n and s = 2, 3, .. , k where n is the number of samples, p is the number of covariates in the model, and k is the number of categories of response variable.

Defining the parameters as above, the hierarchical representation of the model is as follows. $l_{i}|\lambda_{i},\theta \sim N\left(w_{i}^{T}\theta ,\frac{1}{{\text{Λ}}_{i}}\right),\;{\text{Λ}}_{i}\sim Gamma\left(\frac{\upsilon }{2},\frac{\upsilon }{2}\right),\theta_{j}\sim N\left(0,\tau_{j}\right),\tau_{j}\sim Exp\left(\frac{\lambda_{j}^{2}}{2}\right),\lambda_{j}\sim Gamma\left(\rho ,\;\delta \right),\rho \sim \frac{c}{{\left(\text{1}+c\rho \right)}^{\text{2}}},$$\delta \sim \frac{c\prime }{{\left(\text{1}+c\prime \delta \right)}^{\text{2}}}$ and we put non-informative uniform prior on  υ. Using the above mixture representation for the parameters and defining the prior distributions, we obtain following conditional posteriors that lead to a straightforward Gibbs sampling algorithm as outlined in Figure 1.

$$l_{i}|\text{Ω}\sim DTN\left(w_{i}^{T}\theta ,\frac{1}{{\text{Λ}}_{i}}\right)$$

In formula above, DTN stands for doubly truncated normal distribution with mean $w_{i}^{T}\theta$ and variance $\frac{1}{{\text{Λ}}_{i}}$ and Ω represents vector of model parameters plus data. For observation 'i' with *y_i _*= *r, l_i _*must be sampled from normal distribution defined above truncated between *γ_r _*and *γ*_*r*+1 _in each iteration of the algorithm.

$$\theta |\text{Ω}\sim MVN\left({\left[W^{T}\text{Λ}W+T^{*}\right]}^{-1}W^{T}\text{Λ}L,{\left[W^{T}\text{Λ}W+T^{*}\right]}^{-1}\right)$$

The normal distribution defined above is a multivariate normal distribution with mean vector and covariance matrix as specified. In the above equation, $T^{*}=diag\left(\tau_{1}^{-\text{1}},\;...,\;{\tau_{p}}^{-\text{1}}\right),\;\text{Λ}=diag\left({\text{Λ}}_{\text{1}},\;...,{\text{Λ}}_{p}\right)$, *W *is the n*p matrix in which *w_ij _*represents expression level of gene *j *in *i^th ^*sample, *p *is number of genes (variables) in the model, ***L ***= [*l*_1_*, l*_2_*, ..., l_n_*]*T *, and *n *is the number of samples.

$${\tau_{j}}^{-1}|\text{Ω}\sim Inv-Gaussian\left(\sqrt{\frac{\lambda_{j}^{2}}{\theta_{j}^{2}},}{\lambda_{j}}^{2}\right)$$

Inv-Gaussian denotes inverse Gaussian distribution with location $\sqrt{\frac{\lambda_{j}^{2}}{\theta_{j}^{2}}}$ and scale $\lambda_{j}^{\text{2}}$. In each iteration of the Gibbs sampling, each *λ_j _*and Λ*_j _*is sampled from the following fully conditional posterior distributions respectively.

$$\lambda_{j}|\text{Ω}\sim Gamma\left(\rho +\text{1},|\theta_{j}|+\delta \right)\;;j=\text{ 1},\;..,p$$

$${\text{Λ}}_{r}|\text{Ω}\sim Gamma\left(\frac{\upsilon +1}{2},\frac{1}{2}\left[{\left(l_{r}-w_{r}^{T}\theta \right)}^{2}+\upsilon \right]\right);r=\text{1},\;..,\;n$$

The fully conditional posterior distributions for  υ, *u*_1_, and *u*_2 _are proportional to [24]:

$$\begin{array}{c} \upsilon |\text{Ω}\alpha \left[\overset{n}{\underset{i=1}{\prod }}{\text{Λ}}_{i}^{\frac{\upsilon }{2}-1}\text{exp}\left(\frac{-\upsilon {\text{Λ}}_{i}}{2}\right)\right]*\left[\overset{n}{\underset{i=1}{\prod }}\left(\frac{{\frac{\upsilon }{2}}^{\frac{\upsilon }{2}}}{\Gamma \left(\frac{\upsilon }{2}\right)}\right)\right]\\ u_{1}|\text{Ω}\alpha {\left(\frac{1-u_{1}}{cu_{1}}\right)}^{p}*\overset{p}{\underset{j=1}{\prod }}{\left(1+\frac{\left|\theta_{j}\right|}{\delta }\right)}^{-\left(\frac{1-u_{1}}{cu_{1}}+1\right)}\\ u_{2}|\text{Ω}\alpha {\left(\frac{c\prime u_{2}}{1-u_{2}}\right)}^{p}*\overset{p}{\underset{j=1}{\prod }}{\left(1+\frac{c\prime u_{2}}{1-u_{2}}|\theta_{j}|\right)}^{-\left(1+\rho \right)} \end{array}$$

As we can see, the fully conditional distributions of  υ, *u*_1_, and *u*_2 _do not have closed form and thus we adopt the following embedded griddy gibbs sampling to sample from them [19,24]. On a grid of *k *values (υ_1_, υ_2_*, ...*, υ*_k _*) representing the degrees of freedom we consider, we perform the following procedure:

• Calculate the weights as *r_i _*= *π*(υ*_i_|−*) using fully conditional posterior obtained for υ.

• Normalize the weights $r_{i}^{N}=\frac{r_{i}}{\sum_{i=1}^{k}r_{i}}$

• Sample one value from (υ_1_, υ_2_*, ...*, υ*_k_*) with probabilities $\left(r_{\text{1}}^{N},r_{\text{2}}^{N},\;...,\;r_{k}^{N}\right)$. On a grid of values in interval (0, 1) we use the same procedure to sample one value from *u*_1 _and *u*_2 _to use in the current iteration of Gibbs sampling. The only difference is that at the end of the procedure we transform *u*_1 _and *u*_2 _back to *ρ *and *δ *using $\rho =\frac{1}{c}\left[\frac{1}{u_{1}}-1\right]$ and $\delta =\frac{1}{c\prime }\left[\frac{1}{u_{2}}-1\right]$ respectively. In the case of ordinal multinomial response, we assign independent uniform priors to thresholds and the fully conditional posterior distribution for thresholds is a uniform distribution and we sample them in each iteration of Gibbs sampling alongside other parameters in the model [29].

$$\gamma_{s}|\text{Ω}\propto \overset{n}{\underset{i=1}{\prod }}\left[I\left(y_{i}=s-\text{1}\right)*I\left({\gamma_{s-}}_{\text{1}}\leq l_{i}<\;\gamma_{s}\right)+I\left(y_{i}=s\right)*I\left(\gamma_{s}\leq l_{i}<{\gamma_{s}}_{+\text{1}}\right)\right]$$

The conditional posterior distribution of *γ_s _*can be seen to be *Uniform*(*δ*_1_*, δ*_2_) in which *δ*_1 _= *max*[*max_i_*[*l_i_|y_i _*= *s − *1], *γ*_*s−*1_] and *δ*_2 _= *min*[*min_i_*[*l_i_|y_i _*= *s*]*, γ_s_*]. It should be noted that *I*() is the indicator function and its value is one if its argument is true and is zero otherwise [29].

### Dataset and Feature Selection

The method was applied to a published dataset on prostate cancer progression downloaded from Gene Expression Omnibus at NCBI (GSE6099) [28]. The dataset contains gene expression values for 20,000 probe sets and 101 samples corresponding to five prostate cancer progressive stages (subtypes): Benign, prostatic intraepithelial neoplasia (PIN), Proliferative inflammatory atrophy (PIA), localized prostate cancer (PCA), and metastatic prostate cancer (MET) [28]. Since there were only two samples for PIA, we removed these samples from further analysis. Sample accession number and tumor types are listed in Additional file 1. Probes with null values in more than 10% of the samples were removed from the dataset. For the remaining probes, the null values were imputed by using the mean value of the probe across samples with non-null values. Before applying our model to this dataset, for each gene we performed logistic regression for ordinal response. This method enables us to take into account the ordinal nature of the response variable in the analysis and preparation of a gene list used as input to the model. Genes were ranked based on the p-value associated with the hypothesis *H*_0 _: *θ_i _*= 0 from the most significant to least significant. Here *θ_i _*is the parameter associated with gene i. We performed Benjamini and Hochberg FDR correction [32]. An FDR cut-off value of 0.05 resulted in a list of 398 genes. Thus, the input to our model was 398 variables (genes) for 99 samples corresponding to four different prostate cancer subtypes (Additional files 1 and 2). The Gibbs sampling algorithm was implemented in R software and the program ran for 60*k *iterations and the first 20*k *was discarded as burn-in.

### Simulation and Cross-validation Procedure

The dataset was randomly divided into training (N = 50) and test (N = 49) groups so that each group contained an equal number of prostate cancer subtypes Benign, PIN, PCA and MET. Genes were ranked based on the posterior mean of parameters and the top 10 or 50 genes obtained from the model were used for classification. In order to make the model more robust we performed 50 re-samplings on the selection of training and test groups and re-ran the model. Sample accession numbers for training and test sets for each of the 50 runs are listed in Additional file 3. The average performance of SBGG was compared to three well-known classification methods: Support Vector Machine (SVM), Random Forest, and the Sparse Bayesian Generalized Linear Model obtained by imposing double exponential prior (SBGDE) on parameters that we developed previously [27]. SVM was implemented in R software using Kernlab library [33]. Specifically, *ksvm* (*y., data *= *dataset, kernel *= "*rbf dot*"*, type *=′ *nu − svc*′*, prob.model *= *T RU E, kpar *=′ *automatic*′) with automatic sigma estimation was used to fit SVM model. The Random Forest was implemented in R using default parameters in randomForest library [34], We implemented the SBGDE according to [25,27] in R software.

## Results

**Figure 2:**
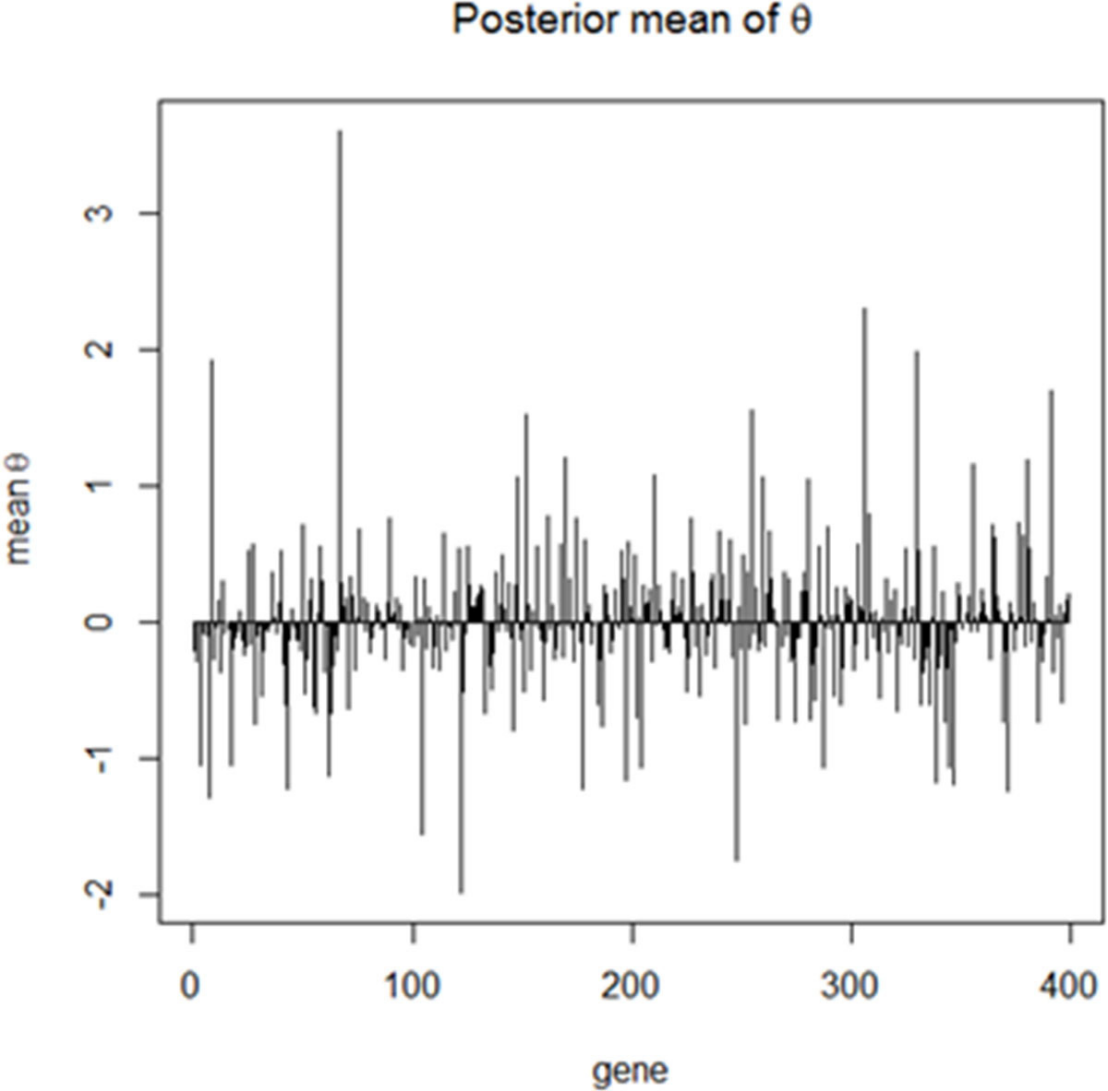
**Posterior mean of *θ*s associated with gene 1 to gene 398**. The x-axis represents the list of 398 differentially expressed genes obtained after Benjamini and Hochberg FDR correction of the results of single gene analysis using classical multi-category logistic regression. The y-axis represents the posterior mean of *θ *associated with each gene. While some signals are reduced toward zero, other signals stand out which turn out to be biologically more relevant to prostate cancer progression subtypes.

We derived the fully conditional posterior distributions for all parameters in a multi-level hierarchical model in order to perform the fully Bayesian treatment of the problem. The Gibbs sampling algorithm was used to estimate all the parameters of the model [35,36], taking into account the progressive levels of the response variable. The top 398 genes ranked base on p-values obtained in initial feature selection step were used as input to our model. The posterior mean of *θ*s for each gene is represented in Figure 2. This result shows that there is no relationship between ***θ ***and the p-value ranking from the initial feature selection methodology.

**Figure 3:**
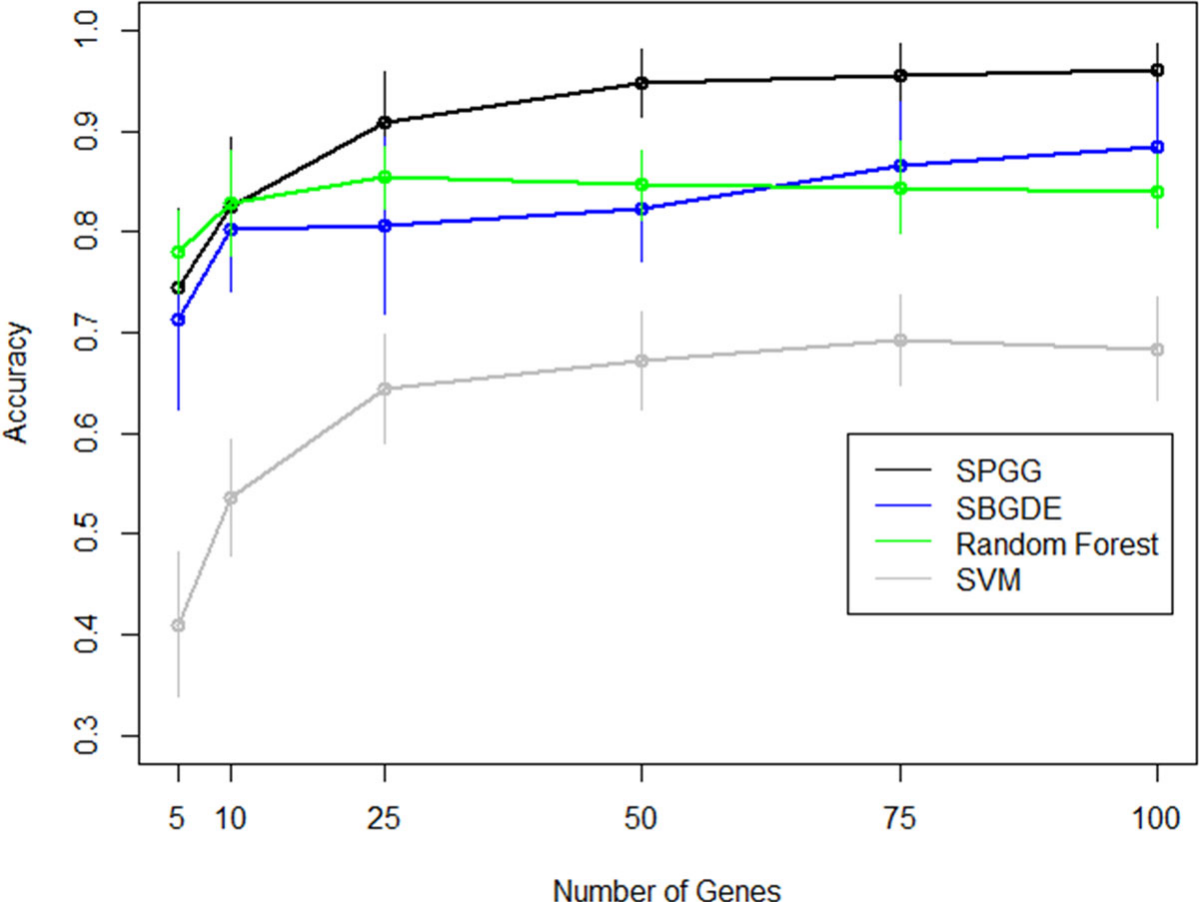
**Accuracy plot of four models using different number of genes for classification of prostate cancer subtypes**. The accuracy values are the average classification accuracy across 50 runs and the vertical lines show their associated standard deviations.

We used the top 50 genes to test the classification accuracy of the SBGG on 50 resampled training and test groups. In order to have a balanced dataset, each training and test group had an equal number of the four prostate cancer subtypes: benign, prostatic intraepithelial neoplasia (PIN), localized prostate cancer (PCA), and metastatic prostate cancer (MET). We found that the average overall classification accuracy of the SBGG model was 94.2% when using 50 marker genes (Table 1). The performance of SBGG model was substantially better than SVM and SBGDE, but was comparable to Random Forest classifier. Next, we examined the performance of SBGG model with regard to classifying the different subtypes of prostate cancer in comparison to SVM, Random Forest, and SBGDE (Table 2). SBGG outperforms SBGDE, and SVM in correctly classifying all sample subtypes and outperforms random forest in all categories except benign by a narrow margin. From a clinical stand point, it is extremely valuable to be able to correctly identify pre-metastatic stages of prostate cancer (PIN, PCA). SBGG performs better than the other three methods in correctly identifying pre-metastatic stages of prostate cancer (Table 2). Also for clinical purposes, it is desirable to be able to perform correct classification based on a smaller number of marker genes. Average classification accuracy of SBGG was 82.5 when using 10 marker genes which was only 0.5% lower that random forest, the closest competitor (Table 1). Additionally, using only 10 marker genes, SBGG outperforms the other three methods in correctly classifying pre-metastatic stages of prostate cancer, which demonstrates consistent performance of the model across different number of marker genes (Table 3). Figure 3 represents the average classification accuracy of all four models using 5, 10, 25, 50, 75, and 100 genes. SBGG classification accuracy is slightly lower when using 5 marker genes compared to random Forest. However, SBGG outperforms the other three methods when using 25, 50, 75, and 100 markers genes for classification.

**Table 1:** Overall average accuracy and associated standard deviations (in parentheses) of SBGG, SBGDE, SVM and Random Forest models using 10 and 50 marker genes

Model	P-10	P-50
SBGG	82.5 (6.8)	94.9 (3.08)
SBGDE	80.4 (6.2)	82.3 (6.4)
SVM	53.6 (5.7)	67 (4.9)
Random Forest	83 (5.2)	84.6 (3.5)

**Table 2:** Average classification accuracy and associated standard deviations (in parentheses) of prostate cancer subtypes in the test group using SBGG, BBGDE, SVM and Random Forest models for 50 marker genes

Sample Type	SBGG	SBGDE	SVM	Random Forest
Benign	95.4 (3.07)	99.6 (1.9)	90.1 (1.7)	96.8 (1.3)
PIN	80.6 (0.08)	53.4 (1.4)	38.2 (8.2)	52 (1.1)
PCA	98.9 (1.9)	65.4 (7.2)	45.8 (6.2)	84.8 (5.4)
MET	96.8 (4.6)	95.4 (6.3)	81.8 (1.6)	83.6 (7.09)

**Table 3:** Average classification accuracy and associated standard deviations(in parentheses) of prostate cancer subtypes in the test group using SBGG, BBGDE, SVM and Random Forest models for using 10 marker genes

Sample Type	SBGG	SBGDE	SVM	Random Forest
Benign	89.4 (6.1)	95.1 (6)	84.4 (5.3)	91.1 (4.5)
PIN	62.5 (1.6)	61.7 (2.8)	9 (7.2)	61.4 (1.9)
PCA	98.7 (0.7)	86.9 (1.1)	37.4 (9)	86.7 (2.1)
MET	59.4 (2.06)	56 (3.2)	55.3 (1.2)	82.8 (7.3)

We next asked if SBGG gene rankings were more or less relevant to the biological mechanisms associated with prostate cancer progression. In order to evaluate the biological relevance for the top ranked genes in the models, we used a literature based method called GeneSet Cohesion Analysis Tool (GCAT) [37]. GCAT is a web-based tool that calculates the functional coherence p-values of gene sets based on latent semantic analysis of Medline abstracts [37-39]. Table 4 shows the average GCAT literature derived p-values (LPv) for the top 100 genes obtained from 50 runs of SBGG, Random Forest, and SBGDE. In addition, we compared the average functional cohesion of the top 100 genes produced by SBGG to the top 100 genes ranked by single gene analysis p-values obtained by ordinal logistic regression. We found that, on average, SBGG produced more functionally cohesive gene lists (LPv = 2.0E-4) compared to SBDE (LPv = 0.007), ordinal logistic regression (LPv = 0.047) and Random Forest (LPv = 0.131). Notably, 100% of the SBGG runs had smaller LPv than 0.047, which was produced by ordinal logistic regression using single gene analysis. The literature p-value for the median run of SBGG was 4.50E-06 compared to 1.90E-04 for SBGDE and 2.85E-02 for Random Forest. Thus, while Random Forest was the closest competitor to SBGG in terms of classification accuracy, the genes obtained from Random Forest are less biologically relevant. Based on these results, we conclude that SBGG produces higher classification accuracy than other methods, and identifies more biologically relevant gene markers.

**Table 4:** Literature based functional cohesion p-values (LPv) and associated standard deviations (in parentheses) of the top 100 genes obtained from SBGG, SBDE, logistic regression, and Random Forest models

Sample Type	Lpv
SBGG	2.0E-4 (1.7E-5)
SBGDE	0.007 (0.001)
Ordinal Logistic Regression	0.047
Random Forest	0.131 (0.07)

## Discussion

Microarray gene expression technology is commonly used to gain insights into the mechanisms of human disease and to develop classifiers for prediction of outcomes [40,41]. Gene expression based classifiers can be used for diagnosis of disease as well as for specifically tailoring treatments for individuals [42,43]. Developing robust classifiers is hampered because gene expression experiments measure thousands of genes across a few number of samples, known as the "large p, small n" situation in statistical modeling. Previous studies have shown that the correct selection of subsets of genes from microarray data is important for accurate classification of disease phenotypes, [44,45]. However, statistical classifiers are prone to over-fitting to the specific cohort under investigation and may not be generalizable to other cohorts [46,47]. From a biological perspective, classifiers are more generalizable if they focus on specific pathways that are mechanistically related to the disease phenotype. In this study, we have developed a sparse Bayesian generalized double pareto model which addresses the "large p, small n" problem and produces a more functionally cohesive set of genes.

The Generalized Double Pareto (GDP) prior distribution was proposed, in linear regression framework, as an alternative to induce sparseness in situations when we are faced with large number of variables compared to sample size [24]. This prior has a simple analytic form, yields a proper posterior and possesses appealing properties, including a spike at zero, Student t-like tails, and a simple characterization as a scale mixture of normals leading to a straightforward Gibbs sampler for posterior inferences that makes Bayesian shrinkage estimation and regularization feasible [24]. Utilizing this prior in a more general framework of generalized linear models, we presented a Bayesian hierarchical model to handle multi-category outcome situations when the number of variables is much larger that sample size. While shrinking small effects toward zero and producing sparse solutions, the over shrinkage problem caused by using light-tailed priors is remedied by the heavier tails obtained via mixing over the hyper parameters [24].

We used the Sparse Bayesian Generalized Linear Model (SBGG) model to do prediction of tumor type on the test dataset. We showed that the average classification accuracy of SBGG using 50 marker genes was substantially higher than other competing methods. In clinical applications, it is desirable to reduce the number of marker genes and be able to perform predictions based on a smaller set of markers. Using ten marker genes, average classification accuracy using SBGG was higher than SVM and SBGDE and slightly lower (0.5%) than random forest. It is important to note that SBGG performs substantially better in correctly identifying premetastatic (PIN, PCA) stages of prostate cancer which can prove extremely useful for diagnostics and therapeutics in clinical settings. SBGG substantially outperforms the other 3 methods in correctly identifying pre-metastatic stages of prostate cancer regardless of the number of marker genes utilized for prediction purposes.

As seen in Figure 3, SVM performance is lower than the other three methods. In multi-class classification with *k *categories, "ksvm" uses one-against-one approach in which $\frac{k\left(k-1\right)}{2}$ binary classifiers are trained. The appropriate class is found by a voting scheme. The class that gets maximum votes is the winning class. In this paper, we declared a winning class when votes exceeded 50%, which is quite stringent. After closer examination, we found that in some cases SVM identified the correct class, but the number of votes was below the 50% threshold. This result indicates that SVM is less sensitive than the other methods.

Importantly, SBGG identified more biologically relevant gene sets in addition to showing better classification performance (Table 4). This result indicates that by having heavier tails in the prior distributions, SBGG is able to identify weaker gene expression changes that have more functional relevance to the phenotype of interest. Thus, we posit that SBGG may be a better approach to simultaneously identify marker genes for classifications as well as gaining insights into the molecular mechanisms of the phenotype under investigation.

It is important to note that the classification accuracy of all three models were compared using a selected set of 398 genes which were obtained based on p-value of a single gene analysis using an ordinal regression model. Hence, this may bias the initial gene selection process. It is possible that some biologically relevant genes to the prostate cancer progression might have been missed by this analysis due to low signal. One way to perform an initial gene selection could be to consider gene pathway information as described previously by others [48]. Our future plan is to evaluate SBGG performance using pathway driven feature selection methods while considering more complex covariance matrix structure which takes into account gene-gene interactions. Also, we plan to incorporate literature information into the prior distributions in order to design literature informed priors that would potentially enable us to obtain machine learning models with high classification accuracy which provide a very enriched set of markers with high biological relevance to the phenotype under study.

## Competing interests

The authors declare that they have no competing interests.

## Authors' contributions

Study Design: B.M, L.Y.D, R.H

Model Development: B.M, D.B, L.Y.D

Analysis: B.M, S.R

Manuscript Preparation: B.M, R.H

## Supplementary Material

Additional File 1 **Samples**. This excel file named samples.xlsx contains the sample accession numbers and and tumor type for all 99 samples. Click here for file

Additional File 2 **Input gene list**. This excel file named InputGeneList.xlsx contains the list of 398 genes obtained after Benjamini and Hochberg FDR correction. Click here for file

Additional File 3 **Train and Test samples-50 runs**. This excel file named RunDetails.xlsx contains accession number of samples randomly selected for training and testing. Click here for file
